# Clinical presentation of strokes confined to the insula: a systematic review of literature

**DOI:** 10.1007/s10072-021-05109-1

**Published:** 2021-02-11

**Authors:** Vincenzo Di Stefano, Maria Vittoria De Angelis, Chiara Montemitro, Mirella Russo, Claudia Carrarini, Massimo di Giannantonio, Filippo Brighina, Marco Onofrj, David J. Werring, Robert Simister

**Affiliations:** 1 Department of Biomedicine, Neuroscience and advanced Diagnostic, University of Palermo (BIND), Palermo, Italy; 2 Stroke Research Centre, Institute of Neurology, UCL, London and The National Hospital for Neurology and Neurosurgery, University College London Hospitals NHS Trust, London, UK; 3 Department of Neurology, SS Annunziata Hospital, Chieti, Italy; 4 Department of Neuroscience, Imaging and Clinical Science, G. d’Annunzio University, Chieti, Italy

**Keywords:** Insular stroke, Insular dysfunction, Insular syndromes, Cerebrovascular disease

## Abstract

**Background and purpose:**

The insular cortex serves a wide variety of functions in humans, ranging from sensory and affective processing to high-level cognition. Hence, insular dysfunction may result in several different presentations. Ischemic strokes limited to the insular territory are rare and deserve a better characterization, to be quickly recognized and to receive appropriate treatment (e.g. thrombolysis).

**Methods:**

We reviewed studies on patients with a first-ever acute stroke restricted to the insula. We searched in the Medline database the keywords “insular stroke” and “insular infarction”, to identify previously published cases. Afterwards, the results were divided depending on the specific insular region affected by the stroke: anterior insular cortex (AIC), posterior insular cortex (PIC) or total insula cortex (TIC). Finally, a review of the clinical correlates associated with each region was performed.

**Results:**

We identified 25 reports including a total of 49 patients (59.7 ± 15.5 years, 48% male) from systematic review of the literature. The most common clinical phenotypes were motor and somatosensory deficits, dysarthria, aphasia and a vestibular-like syndrome. Atypical presentations were also common and included dysphagia, awareness deficits, gustatory disturbances, dysautonomia, neuropsychiatric or auditory disturbances and headache.

**Conclusions:**

The clinical presentation of insular strokes is heterogeneous; however, an insular stroke should be suspected when vestibular-like, somatosensory, speech or language disturbances are combined in the same patient. Further studies are needed to improve our understanding of more atypical presentations.

**Supplementary Information:**

The online version contains supplementary material available at 10.1007/s10072-021-05109-1.

## Introduction

The insula of Reil is a small brain structure, lying in the Sylvian fissure and hidden behind the frontal, parietal and temporal opercula. The insular cortex is considered a “hub” interconnecting several networks [1, 2] and contributing to motor and multimodal sensorial and cognitive functions [3, 4], and our knowledge comes from research using animal models, brain stimulation and functional magnetic resonance imaging (MRI) [1]. In addition, insular function has been the object of several studies in healthy volunteers and in the context of neurodegenerative [5], tumours [6] and cerebrovascular diseases [1]. Focal ischemic lesions restricted to the insular cortex (insular stroke, IS) are rare, because of the frequent involvement of adjacent brain regions sharing common vascular supply [7]. Hence, the majority of lesions reported in the literature that include the insula is represented by large territory strokes due to middle cerebral artery (MCA) occlusion [8]; in these conditions, insular dysfunction is almost always overshadowed by more striking symptoms [9] linked to injury to non-insular brain territories. As a result, the clinical presentation of IS recorded in the literature appears heterogeneous, making it difficult to rapidly reach diagnosis in cases of isolated insular injury and so provide prompt treatment.

The first reports of insular dysfunction secondary to stroke were published some years ago [10, 11]. However, the current evidence remains limited to small case series and case reports [12–15]. This review aims to analyse the available literature in order to characterize the epidemiology, pathophysiology and clinical presentation of acute ischemic strokes in patients with a first event stroke restricted to the insular territory, to highlight atypical manifestations and provide updated information on the incidence and importance of IS.

## Methods

We carried out a systematic review of the literature, through a comprehensive MEDLINE search, in order to identify all available original studies describing isolated insular ischemic lesions. The systematic review was performed according to the PRISMA guidelines. The following search words were used: “insular stroke” and “insular infarction”. The search was conducted on June 9, 2019, and yielded overall 70 records. Further 11 papers were later added following an additional screening of references from unselected papers. We included all original articles (prospective or retrospective observational studies) written in English, in which subjects presented with an isolated insular ischemic lesion. We included studies involving adults only. We excluded 44 records by reviewing article abstracts, and following detailed examination of the full texts of the 37 remaining articles, we found 25 papers meeting our inclusion/exclusion criteria, which were subsequently included in the qualitative synthesis (Fig. I, Supplementary material). The inclusion and exclusion criteria are agreed by all the authors (Supplementary material).

**Fig. 1:**
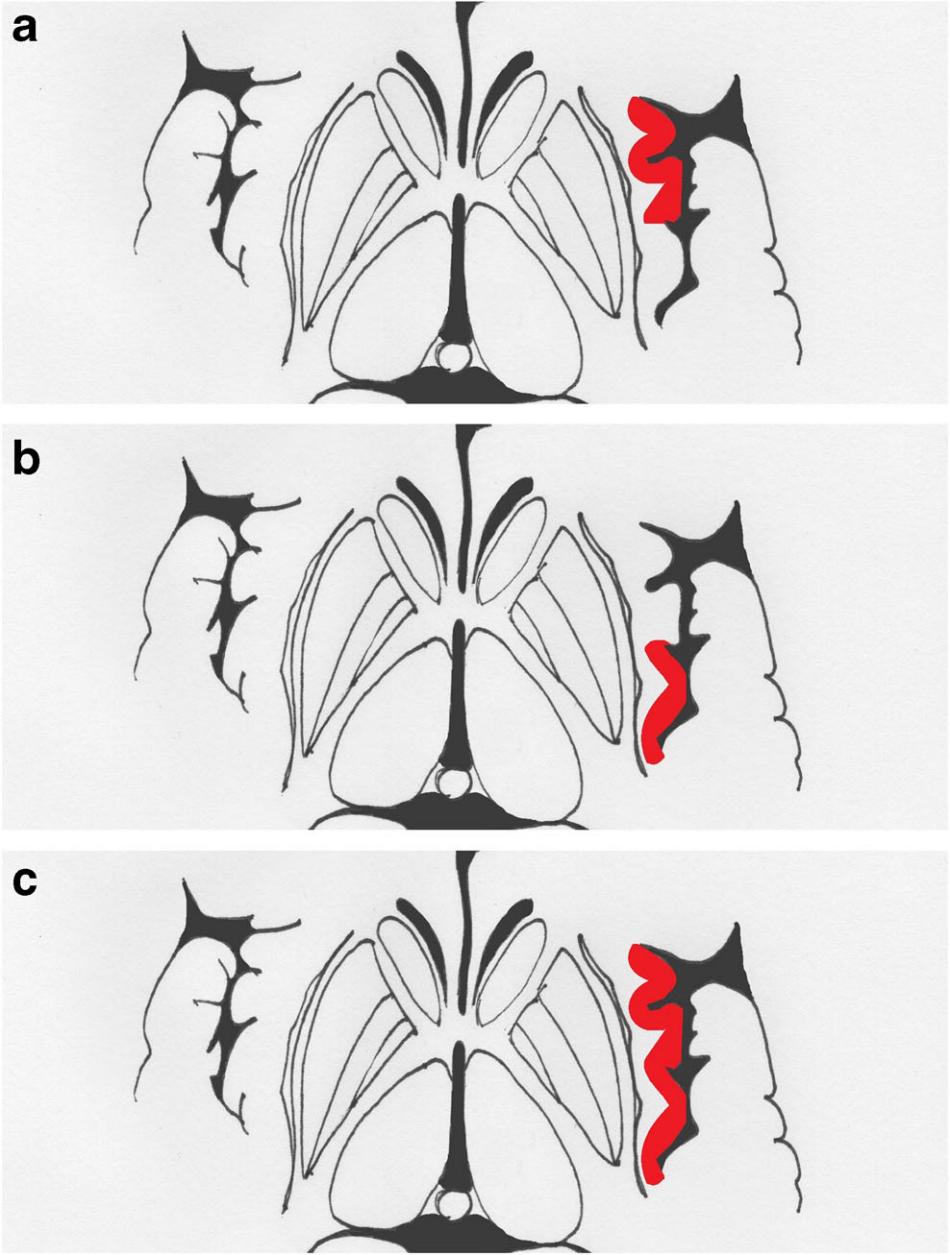
The figure shows the possible site of injury in the insular cortex (red). Ischemic strokes confined to the anterior insular cortex are situated between the frontal lobe and the central sulcus (**a**); the temporal lobe and the central sulcus delimitate the posterior insular region (**b**). The total insular territory can be involved in bigger strokes that overcome the central sulcus (**c**)

We analysed data extracted from case series and case reports focusing on the clinical features of IS. Finally, we assigned patients to one of three groups according to the site of the lesion, as assessed utilizing MRI or computed tomography. The first two groups consisted of patients with ischemic lesion confined to the anterior (AIC, Fig. 1a) or posterior (PIC, Fig. 1b) insular cortex; the third group gathered patients with larger lesions extended to both anterior and posterior insula (total insular cortex, TIC, Fig. 1c). Also, we distinguished right from left IS to better define the role of lateralization on the clinical phenotypes of IS.

We analysed the differences in epidemiology, aetiology according to stroke mechanism as defined with the TOAST classification system [16] and clinical findings among the groups.

## Results

### Characteristics of studies included

The literature search identified 70 papers, and 37 were selected after screening titles and abstracts (Supplementary material). Figure I (Supplementary material) shows a “Prisma Diagram” [17] of studies included and excluded. Out of the 37 retrieved, 9 studies were excluded because they were not focused on insular lesions, 2 because they were reviews and 1 because it reported insular stroke data without focusing on clinical manifestations. Thus, after reviewing the full text, we included 25 papers. Supplementary Table I shows all included studies on insular stroke [10–13, 15, 18–37].

### Patients

**Fig. 2:**
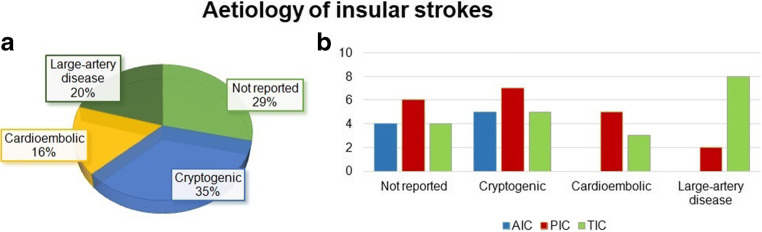
In “**a**”, aetiology is shown among insular strokes. In “**b**”, for each site of lesion (AIC, PIC and TIC), the more common aetiologies are reported. AIC, anterior insular cortex; PIC, posterior insular cortex; TIC, insular stroke that involves both anterior and posterior territory

We found 49 patients (59.7 ± 15.5 years old, 24 males) from literature with strokes confined to the insula (Supplementary Table I) [10–13, 15, 18–37]. Figure 2 shows the distribution of aetiology in all patients and with the site of the lesion.

### Symptoms of insular strokes

**Fig. 3:**
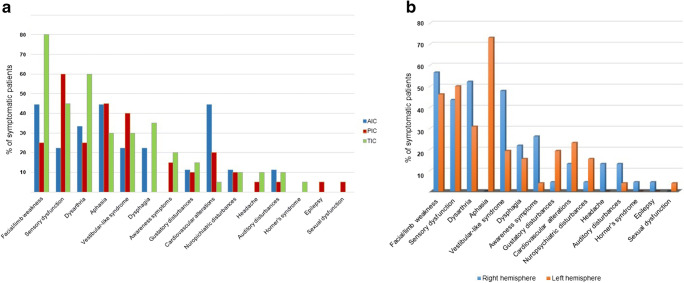
In “**a**”, the most common symptoms of insular stroke are listed; for each symptoms, the frequency is shown in relation to the site of lesion. In “**b**”, symptoms of right and left insular stroke are compared, showing the effect of lateralization

Supplementary Table II and Fig. 3 describe the symptoms of patients with IS, their frequency and relationship with the site and side of the lesion. Motor and sensory deficits, dysarthria, aphasia, vestibular-like syndrome, dysphagia, awareness deficits, gustatory disturbances, cardiovascular alteration and dysautonomia and neuropsychiatric disturbances were the most frequent symptoms. Further unusual symptoms were reported in a minority of cases.

### Site and side of lesions

We found 9 patients (57.8 ± 12.1 years old, 5 males) with strokes in the AIC (18%), 20 patients (57.6 ± 18.6 years old, 8 males) in the PIC (41%) and 20 patients (62.2±13.6 years old, 12 males) in the TIC (41%). Supplementary Table III and Fig. 3a report symptoms and frequency with the site of the lesion.

We found 23 patients with strokes in the right (47%) and 26 in the left (53%) insular cortex. Supplementary Table IV and Fig. 3b report symptoms and frequency in relation to the side of the lesion.

## Discussion

This systematic review summarizes the current evidence on clinical presentation of insular strokes. Ischemic strokes can rarely involve the insula, in the case of a big lesion, but they are even more rarely limited to the insular lobe; in these cases, the infarct may involve the anterior or the posterior cortex and sometimes even the whole insula. We found that strokes limited to the insula are very rare, even if their incidence is probably underestimated. The aetiology is cryptogenic in most cases. This clinical and radiological entity can present with a constellation of symptoms that are an association of typical symptoms of MCA strokes, combined to unusual features that are more connected to the insular dysfunction. Such presentations depend on the site of lesion or lateralization.

### Previous reports

The appearance of symptoms related to insular dysfunction has been the object of several studies in patients affected by stroke from the late 1990 to the last 20 years [10–15]. In one study, 4 cases of IS were reported among 4800 (0.08%) patients with first acute stroke [10]; the authors concluded that clinical presentations of insular stroke fall into five principal clinical presentations: somatosensory, gustatory, vestibular-like, cardiovascular and neuropsychological syndromes. In 2004, a series was published of 11 patients among 2600 patients (0.4%) with first-ever ischemic stroke restricted to subinsular territory between 1999 through 2002 [12]. In this paper, based on a larger sample population, some additional symptoms appeared such as dysphagia, transcortical motor aphasia, dysarthria, neglect and apathy. Subsequently, a retrospective multicentric analysis identified 7 more patients with IS, reporting a similar constellation of symptoms without new features [15]. More recently, a paper reported a higher incidence of IS, describing 10 patients with PIC out a total population of 475 [14]. In this paper, no vestibular-like symptoms were not described.

### Aetiology

Pure insular stroke is very rare; hence, no epidemiological studies have been performed to date. The insula is supplied from the M2 segment of the MCA. Embolic occlusion of M2 or its branches is the main cause of IS, but the exact aetiology and the origin of embolism are debated. From published case series and case reports, the aetiology is usually unknown or not reported (Fig. 2). Large-artery disease seems to be the most prevalent aetiology, especially in the context of larger lesions, followed by cardioembolism (Fig. 2) [12].

In the more recent past, reporting on insular strokes has become more frequent, probably because of successful recanalization rates through thrombolysis and thrombectomy with the understanding that successful thrombolysis or thrombectomy has the potential to fragment the acute occlusive clot, which can migrate downstream, thus occluding smaller branches, such as insular feeders [8]. Finally, the use of sophisticated neuroimaging techniques and high field MRI has allowed a more accurate definition of the insular region and the description of strokes confined to this small territory.

### Clinical manifestations of insular stroke

A combination of somatosensory and speech disturbances usually suggests a complete MCA stroke, but the co-occurrence of rarer symptoms could, instead, suggest a more focal involvement of the insula (Supplementary material) [4, 9]. Although most patients with IS present with symptoms that can be grouped into a small number of symptom, cluster presentations are also described with a wider array of symptoms.

### Weakness and motor deficits

Motor deficits are the most common symptoms encountered in IS, ranging from facial weakness to hemiplegia [11, 12, 15, 24, 32–34, 36]. Hemiparesis is the typical presentation, followed by a facio-brachial pattern of weakness (Supplementary Table II). Motor deficits more often occur in association with other symptoms and are more frequent in lesions involving both the AIC and PIC [11, 12, 15, 24, 32–34, 36]. In fact, weakness was reported in 53% of patients with IS. Moreover, among patients who presented motor deficits, 61% presented a lesion involving the whole insula, 23% in the PIC and only 15% in the AIC (Supplementary Table II).

### Somatosensory deficits

Somatosensory deficits are very common following IS, especially in lesions of the PIC and with more extended lesions (Supplementary Table II, Fig. 3); they range from difficulties in controlling directions of movements to more variable deficits in vibration sense, astereognosis and dysesthesia with positive sensations in absence of stimuli [10, 12, 13, 15, 19, 23, 26, 28, 32, 37]. Sensory symptoms appear to be transient, usually last a few days and then subsiding over the following weeks, but can persist and become chronic. Mild deficits or numbness in the contralateral upper limb are the most frequent sensory complaint and are often extended to involve the contralateral body and face. Rarely, IS may cause dissociated sensory deficits involving a single modality [12, 26, 28] or non-specific sensory complaints [12, 15].

### Dysarthria

Dysarthria is another common manifestation in patients affected by IS [10–12, 15, 23, 24, 32, 33, 35, 36]. It appears with similar rates in both right and left IS and is more frequent in bigger lesions and PIC strokes. Most reports do not quantify the severity of dysarthria, and there is only one report of complete anarthria in a patient with bilateral ageusia and dysphagia [23].

### Aphasia

Aphasia is a typical finding of dominant hemisphere IS, being reported in 73% of left hemisphere IS [10–12, 15, 18, 24, 26, 29, 32, 36]. Language deficits are well-reported ranging from a word-finding difficulty with anomia and mild lexical dysgraphia-dyslexia to more severe non-fluent aphasia with altered comprehension; the most common pattern is non-fluent aphasia with anomia but essentially preserved comprehension and repetition [12, 15]. These deficits may be severe-moderate with phonemic paraphasia (42% of aphasic patients with IS) or mild with word-finding difficulty (21%), but they usually recover in a few days with mild or no residual deficits. Apart from a clear lateralization to the left hemisphere, aphasia seems to be more common in PIC IS (Supplementary Table II).

The high prevalence of aphasia after insular stroke is expected, as it is well-known that a dominant perisylvian MCA infarction (including the insula) often produce motor, sensory or global aphasia [38]. Moreover, distinct aphasic syndromes have been reported depending on the insular topography; according to some authors, lesions in the AIC usually cause Broca’s aphasia, whilst the middle gyri and PIC are associated with conduction aphasia and Wernicke’s aphasia [38].

### Vestibular-like syndrome

A “vestibular-like syndrome” (VLS) has been reported in 33% of IS, representing the fifth most common symptom of IS in order of frequency after sensory-motor deficits and language impairment (Supplementary Table II, Fig. 3) [10, 12, 15, 19, 20, 29, 35]. Moreover, vertigo can be the only symptom of a stroke confined to the PIC [10, 20]. Despite being reported more often after right IS, there is not a clear lateralization for VLS (Supplementary Table II, IV, Fig. 3). VLS have been reported after direct electrical cortical stimulation, but no left-right differences were observed [1, 2]. Similarly, anatomical studies identified the posterior parietal operculum and the retroinsular region as crucial regions for vestibular processing, but, to our knowledge, a major role of the right (respect to the left) insula has not been reported [1, 2]. This difference, not emerging from direct stimulation, might indicate a reduced recovery potential of the right hemisphere from VLS due to right IS. However, this weak evidence should be confirmed and explored from future studies. Overall, VLS is heterogeneous. Patients may present with isolated “vertigo” or “dizziness” with instability. Occasionally, nystagmus and lateropulsion have been also reported, associated with nausea and vomiting [19]. Former studies described dizziness, vertigo, unsteadiness and gait instability without any abnormality of eye movements or nystagmus [12, 15]. Horizontal nystagmus has been reported as an isolated abnormality in a case of IS [29] and described as a “pseudovestibular neuritis”. Subjective dizziness with ataxia has been described in a patient with AIC stroke [31] and possibly explained as occurring as a result of disconnection between supra- and infratentorial centres of balance and coordination [39].

### Dysphagia

Dysphagia is a frequent complaint after IS, being experienced in one out of four patients [11, 12, 15, 23]. Sometimes it is described as coughing or hoarseness after ingestion of liquids, or as non-specific dysphagia. These symptoms appear to be transient with a rapid recovery reported [12]. In our review, 80% of dysphagic patients presented with lesions in the AIC or lesions including the TIC. We found no difference in the incidence of dysphagia depending on the side of the lesion. In a recent paper on ischemic stroke patients, dysphagia was correlated with the region of involvement of the insula [40]. In this study, dysphagia was reported in 22% of TIC strokes and in 40% of AIC strokes but in no patients with PIC injury. On the other hand, AIC was impaired in all patients with dysphagia, thus suggesting the AIC as the cortical region tasked with deglutition control.

### Spatial and awareness deficits

Hemi-spatial and awareness deficits are well-described symptoms resulting from damage to the right hemisphere, and they have also been reported after IS [10–12, 15]. Hemi-spatial neglect is the typical manifestation, with loss of capacity to respond to stimuli contralateral to the lesion [41]; patients may also have the impression of loss of awareness of one side (asomatognosia) or deny ownership of a limb or an entire side of one’s body (somatoparaphrenia) [15]. Also, this kind of disturbances can be associated with transient somatosensory deficits [10, 12, 15]. These deficits are reported more frequently in right-sided IS but are present in only 26% of right IS described in the literature (Supplementary Table II). These data may be an underestimate of the true incidence if screening for spatial deficits after stroke is inadequate [42]. Among patients with hemi-spatial awareness deficits, 57% had injury extended to the whole insula, and 43% had PIC lesions, whilst there are no identified cases with AIC lesions (Supplementary Table II).

### Gustatory dysfunction

Alteration in taste recognition, ranging from heightened taste intensity to bilateral ageusia, is a common feature of IS [10, 12, 13, 15, 23, 26]. Unpleasant taste sensation (parageusia) is, however, uncommon and only reported in a few cases. One patient experienced a persistent flavour of rotten melon after an infarct involving the left AIC [13]. Alteration in food preference (Gourmand syndrome) has not been reported to date [23]. In the literature to date, taste disturbance occurred in 12% of IS but did not lateralise or occur more frequently in any IC pattern of injury. Gustatory disturbanceoccurs more frequently and is more severe after left IS, occurring in 19% of left IS, but only 4% of right IS (Supplementary Table IV); bilateral ageusia was reported in 2 patients after left IS [10, 23] and in one patient after right IS [12]. These reports suggest that the dominant AIC represents a crucial region for the perception of taste. As for hemi-spatial awareness symptoms, gustatory disturbances may well be underreported, because taste assessment is not part of the routine neurological exam.

### Neuropsychiatric disturbances

Neuropsychiatric symptoms have been described in approximatively 10% of patients with IS [12, 32, 34, 35]. Whilst these symptoms, if present, may well significantly influence the outcome of IS, they may be missed or mis-registered because a complete neuropsychiatric assessment is not regularly performed in stroke units [43]. Anergia was reported in patients with strokes involving the right insula [42]. Mood disorders have been reported in two patients following IS: one developed apathy [12], the other complained of emotional flatness and loss of empathy, with increased anxiety and phobia [32]. Impairment in verbal and logical memory has been reported after left and right IS, respectively [43]. Executive function and attention difficulties were also reported in two further cases [34, 35], but studies systematically evaluating psychological profile following IS are not available, to our knowledge.

When psychological symptoms have been recognized in the literature, they have been more frequent after damage in the left insula; for this reason, it has been hypothesized that a disconnection of the dominant insula with the frontal lobe and cingulate cortex may be the origin of NPS [43].

### Cardiovascular alterations and dysautonomia

Several cardiovascular disorders have been associated with IS [10, 11, 15, 24, 30, 33, 35]. Arrhythmias are the more common in this category including paroxysmal ventricular tachycardia, atrial flutter, ST abnormalities and ventricular extrasystoles [11, 33, 35]. Hypertensive bursts are also frequently reported [24, 30]. These symptoms may be associated with concomitant cardiac disease, but some authors have considered them an expression of dysautonomia, because they are often associated with hypertensive bursts, light headedness and pallor [44, 45]. More recently, IS has been associated with ST segment abnormalities, high rates of sinus tachycardia and ectopic beats [46]. In particular, right lesions appear to be associated with paroxysmal short-lasting ventricular tachycardia even in the presence of normal results upon cardiological assessment [33] and left-sided lesions with T wave inversion and a left anterior fasciculus block pattern on electrocardiogram in the absence of coronary heart disease or cardiac pathology [24], with atrial flutter and cardiomyopathy [11] and supraventricular and ventricular extrasystoles with short episodes of ventricular tachycardia [35]. Left insular involvement was associated with an increased risk of an adverse cardiac outcome [47]. Also, insular involvement and higher disability at onset were associated with a greater incidence of autonomic dysfunction post-stroke [48]. Furthermore, one report has suggested that insula dysfunction may be a cause of cerebrogenic sudden death [49].

The overall incidence of these symptoms seems to not differ in relation with site and side of the lesion (Supplementary Table II, Fig. 3), but arrhythmias are more frequently described after left IS. Once again, such symptoms probably have an incidence higher than reported, because they may escape routine evaluation and follow-up. A systematic approach to dysautonomia is needed to define the relationship between the insular cortex and these symptoms.

### Auditory disturbances

Auditory disturbances have been described in 4 patients with stroke confined to the insula; typically with increased sensitivity to sound. One of these patients described the character of the experienced transient auditory hallucinations as being similar to “videogame” noises [34].

### Other symptoms

Headache was described in 3 patients with right IS [15, 34]. Finally, there are single cases reporting Horner’s syndrome in IS related to carotid dissection [15], focal seizures following right IS [37] and sexual dysfunction as a result of drop in libido following left PIC stroke [32].

### Prognosis

There are no data on long-term follow-up for IS, but outcomes in the cases reported were generally good with the majority of the reported cases making a good recovery [10, 12]. In the largest case series, more than 50% of patients completely recovered in 2 days, and all had Modified Rankin Scale score of 0 after 6 months [15]. In contrast, large territory strokes involving a large volume of the MCA and including the insula understandably have a worse prognosis than stroke involving only the insula [8]. Injury in the insular region may be associated with a poor outcome through several mechanisms, ranging from dysautonomia to cardiac complications and infections [47, 49]. In the studied literature, right hemisphere IS involving the insula had a poorer prognosis and were larger when compared to left hemisphere stroke possibly because of a lack in recognition of mild right insular strokes [50].

## Limitations

Insular stroke is a rare and underreported condition. This review summarizes the available information. Most published studies were retrospective and include a non-systematic evaluation of unusual symptoms such as neuropsychiatric and dysautonomic disturbances. In addition, some of the symptoms associated with insular strokes may be subtle or unrecognized as having association with insular injury and underestimated.

Furthermore, as insular stroke is often a consequence of damage of a larger area in the MCA territory, it is possible that sensory-motor symptoms and aphasia or neglect from a wider region of MCA involvement, even transient, may hide neuropsychological and behavioural symptoms. Also, in many patients with stroke limited to the insula, many deficits may be due to poor perfusion of the surrounding cortex, as insular strokes are often due to MCA occlusion.

Further investigation, with a larger patient population and prospective studies, is required to confirm and elucidate the functions of insular cortex.

## Conclusion and clinical implications

The insular cortex serves a wide variety of functions in humans resulting, when affected by diseases, in several different manifestations. The insula is frequently affected by ischemic strokes of the middle cerebral artery, but small strokes limited to the insular territory are very rare. For their rarity and unfamiliar symptoms, they pass often unrecognized, and patients fail to receive the appropriate treatment (e.g. thrombolysis). The clinical presentation of insular strokes is heterogeneous. The most common clinical phenotypes are motor deficits, somatosensory deficits, dysarthria, aphasia and a vestibular-like syndrome. Of interest, many patients with strokes limited to the insula experience atypical and unexpected symptoms that may be neglected by clinicians with delays in the diagnosis and treatment. However, an insular stroke should be suspected when vestibular-like, somatosensory, speech or language disturbances are combined in the same patient. Overall, there remains a paucity of data on insular strokes, and the current evidence is limited to case reports and small studies. The main reason is the difficulty in recruiting enough patients with strategic infarcts in the insular cortex. More studies with a prospective design and involving large numbers of patients are still needed to define the real incidence of insular strokes and to deepen our understanding of their phenomenology.

## Supplementary information

ESM 1 (PDF 259 kb)
