# Correction to: Clinical presentation of strokes confined to the insula: a systematic review of literature

**DOI:** 10.1007/s10072-022-06418-9

**Published:** 2022-09-22

**Authors:** Vincenzo Di Stefano, Maria Vittoria De Angelis, Chiara Montemitro, Mirella Russo, Claudia Carrarini, Massimo di Giannantonio, Filippo Brighina, Marco Onofrj, David J. Werring, Robert Simister

**Affiliations:** 1 Department of Biomedicine, Neuroscience and advanced Diagnostic (BIND), University of Palermo, Palermo, Italy; 2 Stroke Research Centre, Institute of Neurology, UCL, London and The National Hospital for Neurology and Neurosurgery, University College London Hospitals NHS Trust, London, UK; 3 Department of Neurology, SS Annunziata Hospital, Chieti, Italy; 4 Department of Neuroscience, Imaging and Clinical Science, G. d’Annunzio University, Chieti, Italy

**Correction to: Neurological Sciences (2021) 42:1697–1704**

**https://doi.org/10.1007/s10072-021-05109-1**

Originally, the article was published with error. The supplementary material were incorrectly published. This has been updated.

The original article has been corrected.

## Supplementary Information

Below is the link to the electronic supplementary material.

Supplementary file1 (PDF 286 KB)

