# Supplementary material for: Correction to: Clinical presentation of strokes confined to the insula: a systematic review of literature
Source: Neurol Sci. 2022 Sep 22;44(2):785. doi: 10.1007/s10072-022-06418-9 (PMC9842553; doi:10.1007/s10072-022-06418-9)

Supplementary material

Inclusion and exclusion criteria for systematic review

Inclusion criteria

1. Prospective or retrospective observational studies involving adults only, written in English, in which subjects presented with an isolated insular ischemic lesion.
2. Subjects presenting with an insular ischemic stroke on a brain scan (magnetic resonance imaging or at least a computed tomography).
3. Availability of an axial and coronal images to determine the involvement of the anterior, posterior or the whole insula.

Exclusion criteria

1. Review articles.
2. Studies not focused on insular stroke describing patients with very big lesions extended to extra-insular areas (frontal, temporal and parietal lobes).
3. Studies lacking of adequate neuroimages or poor quality.
4. Lack of clinical data to make clinical-radiological correlations.
5. Coexistence of brain lesions, psychiatric and neurological diseases or other confounding factors.

Pathophysiology and mechanisms of clinical symptoms in insular strokes

The Insula of Reil is a small brain structure, lying in the Sylvian fissure and hidden behind the frontal, parietal and temporal opercula. Current consensus regards the insular cortex as a "hub" interconnecting several networks (1,2) and contributing to motor, somatosensory, autonomic, gustatory, auditory, speech, affective and language functions (3,4) and is informed by research using animal models (1).

Weakness and motor deficits

The occurrence of motor symptoms is not related to the involvement of insular cortex itself, but is more a consequence of the extension of the lesion to the corticocapsular motor pathway.

Somatosensory symptoms

The insula has an integrative role in the somatosensory function (5), through an afferent pathway from the parietal cortex to the posterior insula and an efferent pathway that modulates posterior parietal cortex (6). Thermal sensation deficits appear to be more frequent in PIC lesions (7–10), suggesting its relevant role for the perception of hot and cold stimuli. Of interest, contralateral dysesthesia with impaired ability to distinguish between ipsilateral hot and cold stimuli on the body sparing the face was described in two patients with lesions in the PIC (8,9). In a recent report, a stroke in the PIC presented with acute-onset pain, described as "burning or freezing sensations" and associated with thermal sensation deficits and severe allodynia (11).

Dysarthria

Articulation of speech is a complex process that requires an integration of motor and higher cognitive behaviours. Many studies demonstrate the dysfunction of the insular cortex in patients with this kind of deficits (12,13). Moreover, apraxia of speech, a condition in which patients are impaired in their ability to coordinate speech movements (with preserved ability to perceive speech sounds), followed insular involvement in 25 patients (14).

Aphasia

The insular cortex has many connections with language areas, such as frontal operculum, fasciculus arcuatus and temporal lobe (15). Finally, some authors argued that the dominant insular cortex has an important role in the coordination of speech (14). It has been reported that a disconnection between the primary and the supplementary motor cortices may lead to alteration of motor speech (16). On the other hand, connections of the insula with both frontal and temporal lobes are important for verbal fluency (17). Moreover, the initiation of speech depends on appropriate motivation and arousal-activation, as for any initiation behaviour; thus, the reciprocal connections among the anterior insula, the cingulate cortex, amygdale, reticular thalamic nuclei and frontal lobe (15) could probably explain the reason why a speech initiation deficit with anomia and difficulty in repetition are the most common features of aphasia in IS.

Vestibular-like syndrome

Some authors have hypothesized that the PIC may be considered the “primary vestibular cortex”, as homologous of the parieto-insular cortex in monkeys (18). This assumption was later supported by studies on functional MRI (19), but it needs a definite validation employing larger samples of patients.

Recent evidence proves the role of the cerebellum in speech disorders. A recent paper focused on vestibular symptoms of IS in 10 patients with selective damage of PIC without evidence for vestibular failure leading the authors to consider that small PIC lesions might be compensated by mechanisms in neighbouring regions within the cortical vestibular network (20). Another proposed theory is that VLS might emerge as a result of paroxysmal discharges from the PIC similar to the vestibular sensations induced by electrical stimulation of the insula (2), rather than a real loss of function (21).

Dysphagia

The AIC is highly connected with several cortical and subcortical areas linked to swallowing function, such as primary and supplementary motor cortices, thalamic and brainstem nuclei. Moreover, there is evidence that the control of volitional swallowing requires an intact insular cortex (13,22). Swallowing is a complex process that involves the integration of volitional and reflexive behaviours; the insular cortex wields an integrative function between motor and sensory modalities (4,23).

Spatial and awareness deficits

Hence, the role of PIC in the representation of space may be hypothesized. Moreover, the insula is very close to the perisylvian regions and the temporoparietal junction (4,23), which are involved in the mechanisms of acute and chronic spatial neglect. For this reason, bigger lesions more likely cause spatial deficits. As for sensory deficits, HSAD can result from disruption of the afferent and efferent pathway with the parietal lobe in the non-dominant hemisphere (19).

Gustatory dysfunction

Anatomical studies have described the insula as a relay station in the gustative pathway (24). After entering the brainstem at the level of the medulla, the gustative projections synapse in the nucleus of the solitary tract and ascend to the thalamus, and then they arrive at the insular cortex (both AIC and PIC), which is

considered the “gustatory primary cortex” (24). Moreover, lesion studies have identified that injury to the right insula induces ipsilateral taste recognition and intensity deficits, whereas injuries to the left insula produce an ipsilateral deficit in taste intensity, but a bilateral deficit in taste recognition (18,25,26). Finally, more recent studies have confirmed the role of PIC in the gustatory function (27). Hence, the taste information from both sides of the tongue is supposed to pass through the left insula before ascending to associative areas of the brain.

Neuropsychiatric disturbances

Executive function and attention difficulties might be a consequence of the convergent structure of the insular cortex. Hence, these deficits indicate more a phenomenon of diaschisis than a specific dysfunction due to specialized functions themselves (17). Interestingly, a case of “hyper painting” has been described after IS in a patient who started a new artistic behaviour with the tendency to avoid cold colours and an intense pleasure associated with painting with warm colours (28).

Cardiovascular alterations and dysautonomia

Despite a large amount of data in favour of the role of the insular cortex in the control of autonomic functions, the mechanisms involved are still unclear. It has been suggested that cardiovascular changes after right MCA infarction are caused by disinhibition of the insula, which lead to enhanced sympathetic tone (29,30), elevated cortisol and hyperglycaemia (31).

Auditory disturbances

Increased sensitivity to sounds can result from disruption of the modulatory function of insular cortex. The insula may play a key role as a multisensory integration and modulation system (1), leading, when injured, to an altered perception of incoming stimuli.

References

1. Uddin LQ, Nomi JS, Hébert-Seropian B, Ghaziri J, Boucher O. Structure and Function of the Human Insula. Vol. 34, Journal of Clinical Neurophysiology. Lippincott Williams and Wilkins; 2017. p. 300–6.

2. Wynford-Thomas R, Powell R. Navigating the Island of reil: How to understand the insular cortex. Pract Neurol. 2017 Apr 1;17(2):122–6.

3. Ibañez A, Gleichgerrcht E, Manes F. Clinical effects of insular damage in humans. Vol. 214, Brain structure & function. 2010. p. 397–410.

4. Stephani C, Fernandez-Baca Vaca G, MacIunas R, Koubeissi M, Lüders HO. Functional neuroanatomy of the insular lobe. Brain Struct Funct. 2011 Jun;216(2):137–49.

5. Eickhoff SB, Jbabdi S, Caspers S, Laird AR, Fox PT, Zilles K, et al. Anatomical and Functional Connectivity of Cytoarchitectonic Areas within the Human Parietal Operculum. 2010;30(18):6409–21.

6. Dijkerman HC. Somatosensory processes subserving perception and action. 2007;189–239.

7. Baier B, Zu Eulenburg P, Geber C, Rohde F, Rolke R, Maihöfner C, et al. Insula and sensory insular cortex and somatosensory control in patients with insular stroke. Eur J Pain (United Kingdom). 2014 Nov 1;18(10):1385–93.

8. Mak YE, Simmons KB, Gitelman DR, Small DM. Taste and olfactory intensity perception changes

following left insular stroke. Behav Neurosci. 2005 Dec;119(6):1693–700.

9. Cattaneo L, Chierici E, Cucurachi L, Cobelli R PG. Posterior insular stroke causing selective loss of contralateral nonpainful thermal sensation. Neurology. 2007;16;68(3)(Jan):237.

10. Michael GA, Relland S, Borg C, Peyron R, Thomas-Anterion C. A role for the insula in color-induced nasal thermal sensations. Behav Brain Res. 2010;212(1):103–8.

11. Bayat M, Bayat A. Letter to the editor: insular stroke presenting with acute onset of pain. Vol. 265, Journal of Neurology. Dr. Dietrich Steinkopff Verlag GmbH and Co. KG; 2018. p. 1472–3.

12. Flowers HL, Alharbi MA, Mikulis D, Silver FL, Rochon E, Streiner D, et al. MRI-Based Neuroanatomical Predictors of Dysphagia, Dysarthria, and Aphasia in Patients with First Acute Ischemic Strokea. Cerebrovasc Dis Extra. 2017 Jan 27;7(1):21–34.

13. Kumral E, Özdemirkıran T, Alper Y. Strokes in the subinsular territory Clinical, topographical, and etiological patterns. 2004.

14. Dronkers NF. A new brain region for coordinating speech articulation. Nature. 1996;384(6605)(Nov 14):159–61.

15. Shuren J. Insula and aphasia. Vol. 240, J Neurol. 1993.

16. Baier B, zu Eulenburg P, Glassl O, Dieterich M. Lesions to the posterior insular cortex cause dysarthria. Eur J Neurol. 2011 Dec;18(12):1429–31.

17. Julayanont P, Ruthirago D, Detoledo JC. Isolated left posterior insular infarction and convergent roles in verbal fluency, language, memory, and executive function. 2016.

18. Cereda C, Ghika J, Maeder P BJ. Strokes restricted to the insular cortex. Neurology. 2002;59(Dec 24):1950–5.

19. Eickhoff SB, Amunts K, Mohlberg H. The Human Parietal Operculum . II . Stereotaxic Maps and Correlation with Functional Imaging Results. 2006;(February).

20. Baier B, Conrad J, Zu Eulenburg P, Best C, Müller-Forell W, Birklein F, et al. Insular strokes cause no vestibular deficits. Stroke. 2013 Sep;44(9):2604–6.

21. Brandt T, Botzel K, Yousry T, Dieterich M, Schulze S, GroDhadern K. Rotational vertigo in embolic stroke of the vestibular and auditory cortices. Vol. 45, 42 NEUROLOGY. 1995.

22. Daniels SK, Foundas AL. The Role of the Insular Cortex in Dysphagia.

23. Nieuwenhuys R. The insular cortex. A review. [Internet]. 1st ed. Vol. 195, Progress in Brain Research. Elsevier B.V.; 2012. 123–163 p. Available from: http://dx.doi.org/10.1016/B978-0-444-53860-4.00007-6

24. Small DM, Jones-gotman M, Zatorre RJ, Petrides M, Evans AC. A Role for the Right Anterior Temporal Lobe in Taste Quality Recognition. J Neurosci. 1997;17(13):5136–42.

25. Pritchard TC, Macaluso DA, Eslinger PJ. Taste Perception in Patients With Insular Cortex Lesions. Vol. 113, Behavioral Neuroscicnce. 1999.

26. Mathy I, Mj D, Jacquerye P. [Bilateral ageusia after left insular and opercular ischemic stroke]. Vol. 159, Rev Neurol (Paris). 2003.

27. Mazzola L, Royet J, Montavont A, Isnard J. Gustatory and Olfactory Responses to Stimulation of the Human Insula. Ann Neurol. 2017;

28. Thomas-Anterion C, Creac'h C, Dionet E, Borg C, Extier C, Faillenot I, et al. De novo artistic

activity following insular-SII ischemia. Pain. 2010 Jul;150(1):121–7.

29. Oppenheimer S, Cechetto D. The insular cortex and the regulation of cardiac function. Compr Physiol. 2016 Apr 1;6(2):1081–133.

30. Oppenheimer SM, Dm MA, Kedem G, Use WMM. Left-insular cortex lesions perturb cardiac autonomic tone in humans. Vol. 6, Research Paper Clinical Autonomic Research. 1996.

31. Colivicchi F, Bassi A, Santini M, Caltagirone C. Prognostic implications of right-sided insular damage, cardiac autonomic derangement, and arrhythmias after acute ischemic stroke. Stroke. 2005;1710–5.

Supplementary Table I. The table describes all 49 patients with ischemic stroke confined to the insula included in our review of the literature. AIC, anterior insular cortex; PIC, posterior insular cortex; TIC, total insular cortex (TIC is not the sum of AIC and PIC, but it is a group of patients with strokes extended to both regions).

**Study**	**Male (M) / Female (F) Age (Years)**	**Symptoms**	**Aetiology**	**Site of lesion (AIC, PIC or TIC)**
Ahn et al, 2010	F 51	Aphasia and rotational vertigo with nystagmus. Aphasia disappeared during the same day, while nystagmus and vertigo lasted for 9 months.	Not reported	TIC
Bamiou et al, 2006 (3)	F 78	Mild speech difficulty with problems with understanding speech in background noise, localizing sounds, sound discrimination, understanding vocal intonation and inflection, and understanding words when sung. No other cognitive impairment.	Not reported	AIC
Bamiou et al, 2006 (8)	F 78	Slurring dysarthria and left-sided hemiparesis and symmetric mild to moderate high-frequency hearing loss. Difficulty in separating speech from background noise, distinguishing intonation and voice inflections, and understanding text that was being sung. No other cognitive impairment.	Not reported	TIC
Bayat et al, 2018	F 90	Left-sided painful paresthesias (burning sensation) gradually settled during the next few weeks. Left-sided decreasing sensitivity to painful stimuli and impaired ability to distinguish between hot and cold stimuli. Focal left-sided seizures.	Cardioembolic, AF.	PIC
Birklein et al, 2005	M 64	Mild receptive aphasia. Contralateral to the infarct, the patient did not feel cold, cold pain, or pinprick pain. The other sensory modalities were normal.	Not reported	PIC
Bogousslavsky et al, 1997	F 73	Dizziness and rotary vertigo with unsteadiness and falls.	Unknown	PIC
Boucher et al, 2015 (1)	F 28, right-handed	Hemiparesis and aphasia. She also had transient auditory hallucinations in the form of ‘video-game’ noises for a period of three days during her hospitalisation. Persistent discomfort with high-pitched sounds such as whistling. More aware of “buzzing” sound associated with electric razors,	Not reported	PIC

		electric lights, and electricity pylons which seemed amplified and difficult to ignore when present. Unable to separate sounds with concurrent sources e.g., making it difficult to understand the lyrics in a song. No loss of ability to follow the music rhythm or melody. Neuropsychological assessment revealed mainly executive function (planning, inhibition) and attention difficulties, but no major cognitive deficits.		
Boucher et al, 2015 (2)	F 35, right-handed	Severe neck and head pain followed by sudden onset left hemiparesis. Aware of increased sensitivity to sounds (e.g., television, clocks, and children’s voices). Easily distracted by surrounding noise. For instance, she has to turn off the radio when driving a car and finds it very difficult to follow a conversation when, at the restaurant, many people are talking simultaneously. Although she did not notice any change in her ability to follow the rhythm or melody of music, she avoids listening to music as she finds it very disturbing, especially when there are lyrics. Neuropsychological assessment only revealed mild attentional impairments, and the patient complains of increased fatigue and anxiety.	Dissection of the internal carotid artery	TIC
Brandt et al, 1995	F 30	Rotational vertigo with fall to the left, nausea and vomiting. Rapid head movements provoked the symptoms and vertigo was more pronounced in upright position, with considerable unsteadiness of gait. Sensory loss to the left side. All symptoms resolved after a week.	Cardioembolic, AF	PIC
Cattaneo et al, 2007	M 59	Non-painful thermal sensation on the left side of the body sparing the face	Not reported	PIC
Cereda et al, 2002 (1)	F 73, right-handed	Dizziness, non-rotatory vertigo, unsteadiness without nausea, gait difficulty, instability and tendency to fall.	Unknown	PIC
Cereda et al, 2002 (2)	M 69, right-handed	Dizziness, instability, lateropulsion, numb sensation, right hemisensory deficit, mild stereoagnosia and graphestesia. No recognition saline and acidic solutions bilaterally.	Unknown	PIC

		Transient fluent aphasia with paraphasia and dysarthtia.		
Cereda et al, 2002 (3)	F 48, right-handed	Difficulties in controlling directions of movements, no weakness. Right hemisensory deficit, non-fluent aphasia, anomia, phonemic distortion and dysarthria	Large artery disease	PIC
Cereda et al, 2002 (4)	F 75, right-handed	Woke up in the night with the sensation of being touched by a stranger hand, not recognizing her left upper limb. Dizziness, instability. Gait insecure. Hypoestesia left upper extremity, alteration graphestesia and stereognosis. Transient somatoparaphrenia. High BP (205/80).	Unknown	PIC
Daniels et al, 1997 (1)	M 55	Dysarthria, dysphonia and left upper extremity weakness, mild facial asymmetry. Intermittent coughing after ingestion of liquids.	Not reported	AIC
Daniels et al, 1997 (2)	M 63	Dysarthria, dysphonia (articulatory imprecision, low volume, wet hoarseness) and left side weakness/paresis, with inability to walk, anosognosia, hemispatial neglect, left facial palsy. Anterior bolus loss, et phonation, but not cough after ingestion of liquids.	Not reported	TIC
Daniels et al, 1997 (3)	M 50	Atrial flutter and cardiomyopathy, TIA. Inability to speak and right upper extremity weakness, Babinsky. Intermittent wet hoarseness after ingestion of liquids.	Not reported	AIC
Daniels et al, 1997 (4)	M 54	Word-finding difficulty: anomic aphasia with lexical dysgraphia-dyslexia. No swallowing difficulties.	Not reported	PIC
Etgen et al, 2003	M 50	Acute dizziness and unconsciousness. After awaking he noticed weakness of his left side and involuntary throwing-like movements of the left arm and leg. On examination he presented intermittent dystonic movements of the tongue, jerks of the left facial half and ballistic movements of the left limbs with slight coordination difficulties. BP was 160/80 mmHg on the right and 120/80 mmHg on the left arm. All symptoms completely resolved in the next four days without treatment.	Large-artery disease (complete left common carotid occlusion and subclavian artery occlusion)	PIC

Hiraga et al, 2010	M 72, right-handed	A man with a history of atrial fibrillation suffered dysarthria upon awakening in the morning and was admitted to our hospital. On admission, his blood pressure was 166/114 mmHg. He had paretic dysarthria but no aphasia. The patient was diagnosed with cerebral infarction and underwent therapy with intravenous edaravone and anticoagulants. He was discharged with mild dysarthria 14 days after onset.	Cardioembolism	PIC
Julayanont et al, 2016	M 80, right-handed	After waking up non fluent-aphasia, preserved comprehension, dysarthria, facial drooping and right-side weakness. Symptoms resolved after 2 days. MOCA 20/30: memory deficit, impairment in semantic fluency. MRS = 1 at discharge	Unknown	PIC
Kumral et al, 2004 (1)	M 71	Right facial paresis, hemiplegia, numbness, dysarthria	Cardioembolism	TIC
Kumral et al, 2004 (10)	M 71	Right, hemiparesis, numbness	Unknown	TIC
Kumral et al, 2004 (11)	M 53	Right facial and upper limb paresis, numbness, transcortical motor aphasia, dysphagia, vertigo	Unknown	TIC
Kumral et al, 2004 (2)	F 62	Left facial paresis, hemiplegia, dysarthria, dysphagia, anosognosia, vertigo	Unknown	TIC
Kumral et al, 2004 (3)	M 75	Right facial upper extremity paresis, dysphagia, transcortical motor aphasia	Large-artery disease + cardioembolism	TIC
Kumral et al, 2004 (4)	M 66	Left hemiparesis, apathy, numbness, vertigo	Large-artery disease	TIC
Kumral et al, 2004 (5)	F 78	Left facial paresis, hemiplegia, dysarthria, dysphagia, anosognosia, gustatory disturbance (bilateral deficit for sweet and salty).	Large artery disease	TIC
Kumral et al, 2004 (6)	F 59	Left facial paresis, hemiplegia, dysarthria, dysphagia, hemineglect	Cardioembolism	TIC
Kumral et al, 2004 (7)	M 66	Left facial and upper limb paresis, dysarthria	Large artery disease	TIC
Kumral et al, 2004 (8)	F 68	Left hemiparesis, dysarthria, numbness, tactile extinction	Large artery disease	TIC
Kumral et al, 2004 (9)	F 48	Left hemiparesis, numbness, vertigo	Unknown	TIC
Lemieux et al, 2012 (1)	F 34	Headache, dizziness, a brief transient speech deficit, and sudden left arm and leg hypoesthesia. She had the impression that her left side was missing, and touching her	Not reported, smoking, contraceptives, PFO	PIC

		left arm felt like touching someone else. She also had the impression of being “out of reality”. Neurological evaluation 12h after onset of symptoms revealed only mild deficits in vibration detection and proprioception. At follow-up three weeks later, neurological and neuropsychological evaluations disclosed no deficit.		
Lemieux et al, 2012 (2)	F 71, right-handed	Hemibody pinprick sensory deficit, brachiofacial weakness, nonfluent dysphasia with several phonemic paraphasias and diminished comprehension. Sensory examination other than pain perception could not be assessed adequately due to the aphasia. Six days later, the patient presented only slight word-finding difficulties.	Cardioembolic. History of rheumatic fever and mitral valve stenosis, atrial fibrillation	PIC
Lemieux et al, 2012 (3)	F 32, right-handed	Transient episode characterized by sudden nonfluent aphasia (with phonemic paraphasias and spared comprehension), and inability to write. She also described being unable to drink from a glass because her lips would not move properly despite any evidence of facial weakness. Awkwardly, she reported that her symptoms nearly resolved as she started to cry four hours later. At admission, neurological examination was normal.	Unknown	TIC
Lemieux et al, 2012 (4)	M 53, right-handed	Transient episode of isolated word-finding difficulties, with spared comprehension. The deficit lasted 3-4h. At admission the neurological examination was normal.	Unknown. Smoking, previous silent myocardial infarction.	AIC
Lemieux et al, 2012 (5)	F 40, right-handed	Sudden onset of fluctuating non-fluent aphasia (with good repetition and comprehension and few phonemic paraphasias) associated with right hand paresthesia and an unusual sweet taste in the mouth. She also had the impression of a foreign arm (somatoparaphrenia) without misoplegia or anosodiaphoria. Symptoms regressed over the next 24 hours. Upon admission to the stroke unit, the patient had only a slight spinothalamic sensory deficit on the finger of the right hand. Taste was	Unknown	PIC

		normal as was the rest of the neurological exam.		
Lemieux et al, 2012 (6)	M 49	Temporal headache followed by dysarthria and clumsiness of the left hand. He also reported non-rotatory dizziness, which lasted less than 30 minutes. Upon admission to the stroke unit, he had a complete right Horner's syndrome, minimal dysarthria with drift of the left arm. Within 24 hours, all symptoms had remitted.	Large artery disease (internal carotid artery dissection)	TIC
Lemieux et al, 2012 (7)	M 58	Dizziness, lightheadedness and pallor. This was associated with dysarthria and dysesthesia of the face, arm and foot. The major symptoms lasted 30 minutes. Upon admission to the stroke unit, the patient had a very mild left hemibody hypoesthesia more predominant at the left hand. Taste was normal as was the rest of the neurological and neuropsychological examination. The patient was completely asymptomatic after 24 hours.	Cardioembolic. Atrial fibrillation.	PIC
Liou et al, 2010	M 64, right-handed	Dizziness and unsteadiness with marked gait difficulty. Tendency to fall on the left side on tandem gait and walking with increased base. Truncal ataxia.	Unknown	AIC
Mak et al, 2005	M 70, right-handed	Heightened taste intensity (returned normal after 1 week). Any changes in olfaction. Alteration of hot and cold sensations. Peripheral vision. Motor aphasia (at 13 months normal, with occasionally speaking hesitation, literal paraphasia and difficulty in some verbal fluency tasks).	Cardioembolism	TIC
Mandrioli et al, 2004	F 68	Mild right ataxic hemiparesis. Right facial and hypoglossal nerve palsy. Dysarthria. Left anterior hemiblock. Global T wave inversion (disappeared 2 months later). Non- fluent aphasia with phonemic paraphasia, anomia, and with essentially preserved com-prehension and repetition. Blood pressure was 150/100 mm Hg and heart rate (HR) was 94 beats per minute (bpm). Macrocitic anaemia. Hyperhomocysteinaemia.	Unknown	PIC

Markostamou et al, 2015	F 45, right-handed	Transient episode of sudden dizziness, dysarthria, and right facial weakness. A comprehensive neurological examination showed no abnormal focal signs. HOLTER-ECG documented autonomic dysfunction with supraventricular and ventricular extrasystoles along AI with short episodes of ventricular tachycardia. On neuropsychological examination her clinical profile offered no indications of significant affective alterations. Impaired executive functioning, inhibitory and attentional control, mental flexibility and conceptual thinking were demonstrated.	Unknown. The patient had a history of iron-deficiency anemia and smoked one pack of cigarettes per day.	AIC
Mathy et al, 2003	M 70, right-handed	Bilateral agueusia, right hemiphyosethesia. Anarthria, swallowing and sensorial disorders regressed in a few weeks, but taste disturbances led to a 6 kg weight loss. After six months, the symptoms disappeared.	Not reported	TIC
Metin et al, 2007	M 66, right-handed	Increase in secretion of saliva and continuous drive to swallow, no swallowing deficts. Referred discomfort due to an annoying taste of rotten melon (persisting for 15 days). No difficulties in distinguishing tastes. Loss of light touch and pinprick sensation.	Not reported	AIC
Nagao et al, 1999	F 67	Sudden disturbance of speech. The patient was alert, but aphonic, showing oral and lingual apraxia, but she was otherwise neurologically normal. Within 48 hours she fully recovered.	Unknown	AIC
Shuren, 1993	F 59	Right facial weakness. Difficulty in initiation speaking and pronouncing words, aberrant prosody with phonemic and rare semantic paraphasias in speech, errors in reading and writing, impaired comprehension and poor repetition. When angry, language was normal. BP was 160/96 mmHg. After 1 month she had difficulties in speech initiation, but recovered from other deficits.	Unknown	AIC

Thomas-Anterion et al, 2010	F 36, right-handed	Right hemiparesis and dysarthria. The day after, the right hemi- paresia recovered while a right hemianesthesia and partial conduction aphasia persisted. She had a right hemianesthesia to warm, heat and pinprick sensations, including on the right side of the face. She also had chronic, severe neuropathic pain with permanent spontaneous burning sensations, combined with freezing cold sensations and with spontaneous paroxysmal pain including paresthesiae and electrical discharges with a very severe allodynia to skin contact the intensity, mainly on the right upper limb. To protect her hand from these painful sensations, she had a glove on her right hand. Her emotional feeling and emotions were less intense than before and she was unable to feel happy herself, even though she was able to correctly identify an emotionally happy situation. Increased anxiety and phobia. Normal psychological testing. Drop in libido. De novo artistic behaviour with the tendency to avoid cold colours (“hyperpainting”). She had intense pleasure from painting with an additional benefit from warm colours and a dramatic worsening of neuropathic pain during creations with cold colours, especially grey.	Not reported, contraceptives.	PIC
Zini et al, 2012	M 79	Dysarthria, mild facial palsy and arm weakness. Paroxysmal short-lasting ventricular tachycardia	Right internal carotid artery near occlusion	TIC

Supplementary table II. Symptoms of insular stroke. AIC, anterior insular cortex; PIC, posterior insular cortex; TIC, total insular cortex (TIC is not the sum of AIC and PIC, but it is a group of patient with strokes extended to both regions); R, right; L, left; FW, facial weakness; UE, upper extremity; *% occurrence and total patient number.

Symptom *	Details *	Site of lesion*	Side*	Studies
Weakness 53% (26)	-FW 8% (2)<br>- UE 8% (2)<br>-FW+UE 19% (5)<br>-hemiparesis 50% (13)<br>-hemiplegia 15% (4)	AIC 15% (4)<br>PIC 23% (6)<br>TIC 61% (16)	R 54% (14)<br>L 46% (12)	Shuren et al, 1993; Daniels et al, 1997; Etgen et al, 2003; Kumral et al, 2004; Bamiou et al, 2006; Lemieux et al, 2012; Zini et al, 2012; Mandrioli et al, 2004; Thomas-Anterion et al, 2010; Boucher et al, 2015; Julayanont et al, 2016
Sensory dysfunction 47% (23)	-numb sensation in the upper limb 35% (8)<br>-hemisensory deficits 39% (9)<br>-dysesthesia 9% (2)<br>-painful paresthesia with allodynia 9% (2)<br>-Ataxia and hemisensory deficits 9% (2)	AIC 9% (2)<br>PIC 52% (12)<br>TIC 39% (9)	R 43% (10)<br>L 57% (13)	Brandt et al, 1995; Cereda et al, 2002; Kumral et al, 2004; Birklein et al, 2005; Cattaneo et al, 2007; Liou et al, 2010; Lemieux et al, 2012; Bayat et al, 2018; Mathy et al, 2003; Mak et al, 2005; Metin et al, 2007; Thomas-Anterion et al, 2010
Dysarticulation 45% (22)	-dysarthria 95% (21)<br>-anarthria 5% (1)	AIC 13% (3)<br>PIC 32% (7)<br>TIC 55% (12)	R 59% (13)<br>L 41% (9)	Daniels et al, 1997; Cereda et al, 2002; Kumral et al, 2004; Bamiou et al, 2006; Hiraga et al, 2010; Lemieux et al, 2012; Zini et al, 2012; Mathy et al, 2003; Mandrioli et al, 2004; Thomas-Anterion et al, 2010; Markostamou et al, 2015; Julayanont et al, 2016
Language dysfunction 39% (19)	-mild non-fluent aphasia with anomia and minor dysgraphia-dyslexia 21% (4)<br>-transcortical motor aphasia 11% (2)<br>-non-fluent aphasia with paraphasias preserved comprehension 42% (8)<br>-severe non-fluent aphasia with preserved comprehension 5% (1)<br>-conduction aphasia 5% (1)<br>-fluent aphasia with altered comprehension 5% (1)<br>-global aphasia 11% (2)	AIC 21% (4)<br>PIC 53% (10)<br>TIC 26% (5)	R 0<br>L 100% (19)	Shuren et al, 1993; Daniels et al, 1997; Nagao et al, 1999; Cereda et al, 2002; Kumral et al, 2004;Mandrioli et al, 2004; Birklein et al, 2005; Mak et al, 2005; Ahn et al, 2010; Thomas-Anterion et al, 2010; Lemieux et al, 2012; Julayanont et al, 2016
Vestibular-like syndrome 33% (16)	-dizziness, non-rotatory vertigo without instability 50% (8)	AIC 13% (2)<br>PIC 50% (8)<br>TIC 37% (6)	R 69% (11)<br>L 31% (5)	Brandt et al, 1995; Bogousslavsky et al, 1997; Cereda et al, 2002; Etgen et al,

	-rotational vertigo with nystagmus 6% (1)<br>-dizziness with instability, lateropulsion 44% (7)			2003; Kumral et al, 2004; Lemieux et al, 2012; Ahn et al, 2010; Liou et al, 2010; Markostamou et al, 2015
Dysphagia 20% (10)	-intermittent coughing or hoarseness after ingestion of liquids 20% (2)<br>-Bolus loss 20% (2)<br>-Dysphagia 60% (6)	AIC 20% (2)<br>PIC 0<br>TIC 80% (8)	R 50% (5)<br>L 50% (5)	Daniels et al, 1997; Kumral et al, 2004; Mathy et al, 2003; Lemieux et al, 2012
Awareness deficits 14% (7)	-Anosognosia (28%)<br>-Somatoparaphrenia (28%)<br>-hemispatial neglect 44% (3)	AIC 0<br>PIC 43% (3)<br>TIC 57% (4)	R 86% (6)<br>L 14% (1)	Daniels et al, 1997; Cereda et al, 2002; Kumral et al, 2004; Lemieux et al, 2012
Gustatory disturbances 12% (6)	-alteration in taste recognition 50% (3)<br>-positive altered taste sensations 33% (2)<br>-not specified 17% (1)	AIC 17% (1)<br>PIC 33% (2)<br>TIC 50% (3)	R 17% (1)<br>L 83% (5)	Cereda et al, 2002; Kumral et al, 2004; Mathy et al, 2003; Mak et al, 2005; Metin et al, 2007; Lemieux et al, 2012
Cardiovascular alterations and dysautonomia 16% (8)	-arrhythmias 50% (4)<br>-hypertensive burst 37% (3)<br>-lightheadedness and palor 13 % (1)	AIC 37 % (3)<br>PIC 50% (4)<br>TIC 13% (1)	R 37 % (3)<br>L 63 % (5)	Shuren et al, 1993; Cereda et al, 2002; Hiraga et al, 2010; Lemieux et al, 2012; Daniels et al, 1997; Mandrioli et al, 2004; Zini et al, 2012; Markostamou et al, 2015
Neuropsychiatric disturbances 10% (5)	-Mood disorders 40% (2)<br>-Anxiety and phobia 20% (1)<br>-cognitive impairment 40% (2)	AIC 20% (1)<br>PIC 60% (3)<br>TIC 20% (1)	R 20% (1)<br>L 80% (4)	Kumral et al, 2004; Thomas-Anterion et al, 2010; Boucher et al, 2015; Markostamou et al, 2015
Headache 6% (3)	-not specified 8% (3)	AIC 0<br>PIC (1)<br>TIC (2)	R (3)<br>L 0	Lemieux et al, 2012; Boucher et al, 2015
Auditory disturbances 8% (4)	-increased sensitivity to sounds 25% (1)<br>-altered sound recognition 50%<br>-auditory hallucinations 25% (1)	AIC 25% (1)<br>PIC 25% (1)<br>TIC 50% (2)	R 75% (3)<br>L 25% (1)	Bamiou et al, 2006; Boucher et al, 2015
Epilepsy 2 % (1)	-focal seizures (1)<br>-tonico-clinic seizures 0	AIC 0<br>PIC (1)<br>TIC 0	R (1)<br>L 0	Bayat et al, 2018
Sexual dysfunction 2 % (1)	-drop in libido (1)	AIC 0<br>PIC (1)<br>TIC 0	R 0<br>L (1)	Thomas-Anterion et al, 2010
Loss of consciousness 2 % (1)	In a patient with subclavian steal	AIC 0<br>PIC (1)<br>TIC 0	R (1)<br>L 0	Etgen et al, 2003;
Hemiballism<br>2 % (1)	In a patient with subclavian steal	AIC 0<br>PIC (1)<br>TIC 0	R (1)<br>L 0	Etgen et al, 2003;

Supplementary table III. Comparison in symptoms among different sites of lesion in insular strokes. AIC, anterior insular cortex; PIC, posterior insular cortex; TIC, total insular cortex (TIC is not the sum of AIC and PIC, but it is a group of patients with strokes extended to both regions); *% occurrence and total patient number.

**Site of lesion***	**Symptoms ***	**Studies**
AIC 18% (9)	-Weakness 44% (4)<br>-Cardiovascular alterations and dysautonomia 44% (4)<br>-Aphasia 44% (4)<br>-Dysarthria 33% (3)<br>-Dysphagia 22% (2)<br>-Sensory dysfunction 22% (2)<br>-Vestibular-like syndrome 22% (2)<br>-Gustatory disturbances 11% (1)<br>-Neuropsychiatric disturbances 11% (1)<br>-Auditory disturbances 11% (1)	Shuren et al 1993, Daniels et al 1997, Nagao et al 1999, Bamiou et al 2006, Metin et al 2007, Liou et al 2010, Lemieux et al 2012, Markostamu et al 2015
PIC 41% (20)	-Sensory dysfunction 60% (12)<br>-Aphasia 45% (9)<br>-Vestibular-like syndrome 40% (8)<br>-Weakness 25% (5)<br>-Dysarthria 25% (5)<br>-Cardiovascular alterations and dysautonomia 20% (4)<br>-Awareness deficits 15% (3)<br>-Gustatory disturbances 10% (2)<br>-Neuropsychiatric disturbances 10% (2)<br>-Headache 5% (1)<br>-Auditory disturbances 5% (1)<br>-Epilepsy 5% (1)<br>-Sexual dysfunction 5% (1)	Brandt et al 1995, Bogousslavsky et al 1997, Cereda et al 2002, Etgen et al 2003, Mandrioli et al 2004, Birklein et al 2005, Cattaneo et al 2007, Hiraga et al 2010, Thomas-Anterion C et al 2010, Lemieux et al 2012, Boucher et al 2015, Bayat et al 2018
TIC 41% (20)	-Weakness 80% (16)<br>-Dysarthria 60% (12)<br>-Sensory dysfunction 45% (9)<br>-Dysphagia 35% (7)<br>-Aphasia 30% (6)<br>-Vestibular-like syndrome 30% (6)<br>-Awareness deficits 20% (4)<br>-Gustatory disturbances 15% (3)<br>-Neuropsychiatric disturbances 10% (2)<br>-Auditory disturbances 10% (2)<br>-Headache 10% (2)<br>-Cardiovascular alterations and dysautonomia 5% (1)<br>-Horner's syndrome 5% (1)	Daniels et al 1997, Mathy et al 2003, Kumral et al 2004, Mak et al 2005, Bamiou et al 2006, Ahn et al 2010, Lemieux et al 2012, Zini et al 2012, Boucher et al 2015, Julayanont et al 2016

Supplementary table IV. Side of lesions in insular stroke. *% occurrence and total patient number.

**Side of lesion***	**Symptoms**	**Studies**
Right 47% (23)	-Weakness 57% (13)<br>-Dysarthria 52% (12)<br>-Vestibular-like syndrome 48% (11)<br>-Sensory dysfunction 43% (10)<br>-Awareness deficits 26% (6)<br>-Dysphagia 22% (5)<br>-Cardiovascular alterations and dysautonomia 13% (3)<br>-Headache 13% (3)<br>-Auditory disturbances 13% (3)<br>-Neuropsychiatric disturbances 4% (1)<br>-Gustatory disturbances 4% (1)<br>-Horner's syndrome 4% (1)<br>-Epilepsy 4% (1)	Brandt et al 1995, Bogousslavsky et al 1997, Daniels et al 1997, Cereda et al 2002, Etgen et al 2003, Kumral et al 2004, Bamiou et al 2006, Cattaneo et al 2007, Lemieux et al 2012, Zini et al 2012, Boucher et al 2015, Bayat et al 2018
Left 53% (26)	-Aphasia 73% (19)<br>-Weakness 46% (12)<br>-Sensory dysfunction 50% (13)<br>-Dysarthria 31% (8)<br>-Cardiovascular alterations and dysautonomia 23% (6)<br>-Gustatory disturbances 19% (5)<br>-Vestibular-like syndrome 19% (5)<br>-Dysphagia 15% (4)<br>-Neuropsychiatric disturbances 15% (4)<br>-Awareness deficits 4% (1)<br>-Auditory disturbances 4% (1)<br>-Sexual dysfunction 4% (1)	Shuren et al 1993, Daniels et al 1997, Nagao et al 1999, Cereda et al 2002, Mathy et al 2003, Kumral et al 2004, Mandrioli et al 2004, Birklein et al 2005, Mak et al 2005, Metin et al 2007, Ahn et al 2010, Liou et al 2010, Hiraga et al 2010, Thomas-Anterion C et al 2010, Lemieux et al 2012, Boucher et al 2015, Markostamu et al 2015, Julayanont et al 2016

Supplementary Figure I

A “PRISMA” diagram shows the review and screening process in our review of literature.

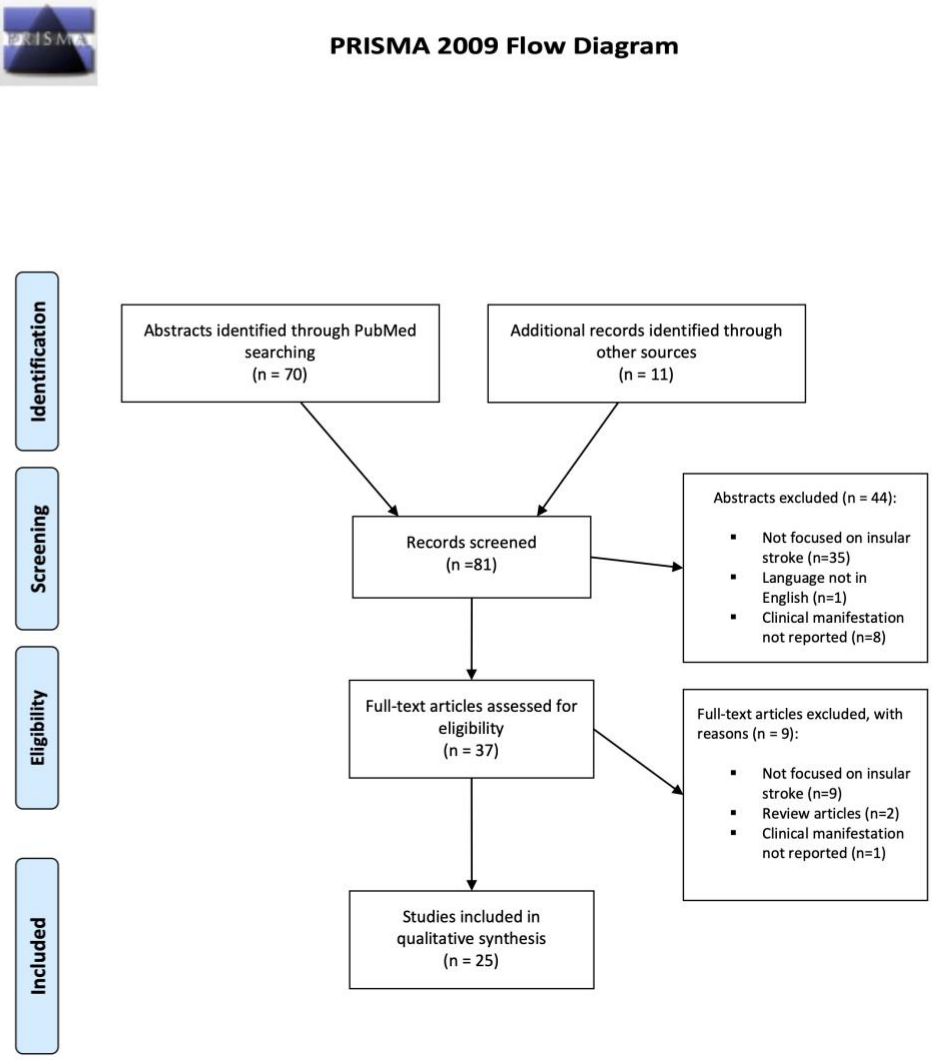

Supplement: Supplementary file 1 — Supplementary file1 (PDF 286 KB) [file 10072_2022_6418_MOESM1_ESM.pdf]
